# Epidemiology of colchicine resistant Familial Mediterranean Fever disease (CrFMF) in Turkey

**DOI:** 10.1186/1546-0096-13-S1-P90

**Published:** 2015-09-28

**Authors:** S Turgay, K Aksu, O Dokuyucu, A Ertenli, A Gul, Y Karaaslan, O Kasapcopur, S Kiraz, AM Onat, H Ozdogan, S Ozen, M Saylan, A Senturk, S Sevgi, S Sezen Cavusoglu, M Tatar, E Tuna, M Turanlı, F Yalcinkaya

**Affiliations:** 1 Novartis Pharma, Medical, Istanbul, Turkey; 2 Ege University, Internal Medicine Rheumatology, İzmir, Turkey; 3 Novartis Pharma, Istanbul, Turkey; 4 Hacettepe University, Ankara, Turkey; 5 İstanbul University, Medical Faculty, İstanbul, Turkey; 6 Ankara Numune Training and Research Hospital, Ankara, Turkey; 7 İstanbul University Cerrahpasa Medical Faculty, Istanbul, Turkey; 8 Hacettepe University Medical Faculty, Ankara, Turkey; 9 Gaziantep University Medical Faculty, Gazantep, Turkey; 10 Ankara University Medical Faculty, Ankara, Turkey

## Introduction

Familial Mediterranean fever disease (FMF) is an autosomal recessively inherited disease characterized by recurrent, self-limited febrile episodes (attacks) with serositis, synovitis, and occasionally skin involvement. The disease primarily affects people of eastern Mediterranean descent, typically presenting at age <20. AA amyloidosis is the most serious complication of FMF and can be life-threatening. Daily colchicine is considered standard of care, and is expected to prevent attacks and amyloidosis in most patients. Turkey has the highest prevalence with 0.1% in general population.

## Objectives

This study aimed at exploring the potential number of FMF patients with colchicine resistance in Turkey.

## Methods

The study was based on expert opinions, as there is currently no defined diagnosis criteria or available data to estimate the patient number. In the first stage of the study, a questionnaire inquiring the type, frequency and duration of the health care resources used for diagnosis and treatment of the disease was prepared. Specific questions were also asked about the epidemiology of the disease and colchicine resistant patients. Ten medical experts were involved in the study. The questionnaire was e-mailed to the experts and median numbers of their answers were calculated. In the next stage, combined answers were discussed in a face-to-face meeting with the experts. All items were discussed one by one until a consensus is reached about the type, frequency and duration of health care resources used during the diagnosis and treatment of FMF patients.

## Results

According to the expert views the prevalence of the disease is 0.1%. Among these patients, 65% are responders to colchicine treatment, 30% are partial responders and 2-5% are colchicine resistant. Colchicine resistant FMF (crFMF) patients are defined by consensus as *patients who have ≥ 1 attacks per month despite maximum tolerable dose of colchicine treatment for 6 months period.*

## Conclusion

Since FMF is highly prevalent in Turkey, there is an unmet need for CrFMF patients. It is estimated that approximately 2.300 CrFMF patients are present in Turkey. This study has also shown that IL-1 agents are used in treatment of CrFMF patients in Turkey.

